# Functional changes of the prefrontal cortex, insula, caudate and associated cognitive impairment (chemobrain) in NSCLC patients receiving different chemotherapy regimen

**DOI:** 10.3389/fonc.2022.1027515

**Published:** 2022-11-02

**Authors:** Siwen Liu, Jie Ni, Fei Yan, Na Yin, Xiaoyou Li, Rong Ma, Jianzhong Wu, Guoren Zhou, Jifeng Feng

**Affiliations:** ^1^ Research Center for Clinical Oncology, Jiangsu Cancer Hospital & Jiangsu Institute of Cancer Research & The Affiliated Cancer Hospital of Nanjing Medical University, Nanjing, China; ^2^ Department of Oncology, Jiangsu Cancer Hospital & Jiangsu Institute of Cancer Research & The Affiliated Cancer Hospital of Nanjing Medical University, Nanjing, China; ^3^ Department of Radiology, Jiangsu Cancer Hospital & Jiangsu Institute of Cancer Research & The Affiliated Cancer Hospital of Nanjing Medical University, Nanjing, China

**Keywords:** non-small cell lung cancer, chemotherapy, cognitive impairment, resting‐state functional magnetic resonance imaging, regional homogeneity

## Abstract

**Introduction:**

Chemotherapy-induced cognitive impairment (CICI), termed “chemobrain”, is highly prevalent in cancer patients following the administration of chemotherapeutic agents. However, the potential pathophysiological mechanisms underlying CICI remain unknown. This study aimed to explore the functional changes of the brain and associated cognitive impairment in non-small cell lung cancer (NSCLC) patients receiving different chemotherapy regimen.

**Methods:**

A total of 49 NSCLC patients (25 patients receiving pemetrexed plus carboplatin chemotherapy (PeCC) and 24 patients receiving paclitaxel plus carboplatin chemotherapy (PaCC)) and 61 healthy controls (HCs) were recruited and underwent resting-state functional magnetic resonance imaging (rs-fMRI) scanning, as well as cognitive function tests including Mini Mental State Exam (MMSE), Montreal Cognitive Assessment (MoCA), Functional Assessment of Cancer Therapy-Cognitive Function (FACT-Cog). Brain functional activities were measured by regional homogeneity (ReHo) values, which were calculated and compared between groups. In addition, the associations between ReHo values of changed brain regions and scores of cognitive scales were evaluated.

**Results:**

NSCLC patients showed decreased scores of MMSE, MoCA and FACT-Cog and decreased ReHo values in the bilateral superior frontal gyrus (medial), middle frontal gyrus, left inferior frontal gyrus (orbital part) and increased ReHo values in the bilateral insula and caudate. Compared with HCs, patients receiving PeCC demonstrated decreased ReHo values in the right superior frontal gyrus (dorsolateral), left superior frontal gyrus (medial orbital), middle frontal gyrus, insula and rectus gyrus while patients receiving PaCC presented increased ReHo values in the right rolandic operculum, left insula and right caudate. Compared with patients receiving PaCC, patients receiving PeCC had decreased ReHo values in the left superior frontal gyrus (orbital part), middle frontal gyrus and increased ReHo values in the left inferior temporal gyrus, lingual gyrus. Moreover, positive relationships were found between ReHo values of the left and right superior frontal gyrus (medial) and the total scores of FACT-Cog in the patient group.

**Conclusion:**

The findings provided evidences that carboplatin-based chemotherapy could cause CICI accompanied by functional changes in the prefrontal cortex, insula, caudate. These might be the pathophysiological basis for CICI of NSCLC patients and were affected by the differences of chemotherapeutic agent administration through different biological mechanisms.

## Introduction

Lung cancer is one of the most commonly diagnosed cancers worldwide with highest incidence rates and highest cancer-related mortality, of which non-small-cell lung cancer (NSCLC) accounts for approximately 80-85% of lung cancer cases (1, 2). Systemic chemotherapy is the primary treatment strategy for patients with NSCLC since most patients have locally advanced, metastatic or unresectable disease at diagnosis (3). Platinum-based chemotherapy (cisplatin or carboplatin regimens) are used to treat a wide variety of cancers and has long been considered as the standard first-line treatment for patients with advanced NSCLC (4). Previous studies have also shown that the survival (both disease-free survival and overall survival) of patients with early-stage NSCLC are improved by curative surgery plus chemotherapy in comparison with surgery alone (5, 6). Although chemotherapy has shown to be beneficial for the treatment of various malignancies, it often causes a series of toxic side effects among which manifestations of central nervous system toxicity are of particular concern (7). The chemotherapy-induced cognitive impairment (CICI), referred as “chemobrain”, is characterized by impaired attention, memory, fatigue, executive functions, processing speed, visuo-spatial skills and behavioural dysfunctions (8). CICI occurring during and after chemotherapy has been found to be associated with neuronal damage, impaired mechanisms responsible for repair and remodelling of brain (9). CICI even persists for a longer period of time with more severe progressive manifestations after discontinuation of chemotherapy (10). CICI is recently becoming more widely recognized, therefore, early identification, aggressive and prompt medical interventions are required to prevent potentially high morbidity and mortality (11).

The occurrence of central nervous system toxicity and its associated CICI are often responsible for impacting long-term quality of life, limiting the dosing regimen, decreasing the effectiveness of chemotherapeutic agents, or even resulting in termination of treatment (12). Therefore, effort should be put forth to explore the mechanisms of CICI to aid in the development of treatments that minimize the behavioral toxicities of chemotherapy, which is vital for long-term survival of cancer patients. Magnetic resonance imaging (MRI), such as structural MRI, functional MRI (fMRI), and diffusion MRI, are the most commonly used and noninvasive methods for measuring functional and structural characteristics of the brain in healthy individuals and patients with psychological or neurological disorders (13, 14). Blood oxygenation level-dependent (BOLD) fMRI, including task-based fMRI and resting-state fMRI (rs-fMRI), has been widely applied to investigate brain function based on the fluctuation of BOLD signal during spontaneous brain activity in specific brain regions when individuals perform explicit cognitive task or during the “resting state” (15). The method of rs-fMRI makes the whole experiment simpler and easier, more reproducible and friendlier to implement when compared with task-based fMRI (16). Numerous longitudinal neuroimaging studies, investigating neurobiological mechanisms underlying CICI, have demonstrated cognitive changes, abnormalities of functional and structural (gray and white matter) brain in cancer survivors treated with chemotherapy (17). While these changes of functional and structural brain partially recover over time after cessation of chemotherapy, subtle changes are still apparent years after chemotherapy in survivors, particularly in long-term survivors (18, 19).

Lung cancer patients exhibited changed static functional connectivity (FC, a measure of brain activity, estimating the spatial distribution of temporal correlations among spontaneous low-frequency fluctuation of different brain regions) pattern in the bilateral dorsolateral prefrontal cortex, which mainly located in the bilateral superior and middle frontal gyrus, left superior temporal gyrus, inferior parietal gyrus, as well as right insula before receiving chemotherapy treatment (20). Decreased dynamic FC variability was found between the right dorsolateral prefrontal cortex and precuneus of lung cancer patients after chemotherapy (20). In addition, altered intrinsic FC pattern within the default mode network (DMN), which reported in patients with mild cognitive impairment as a predictor of Alzheimer’s disease) (21), was found to be associated with CICI of lung cancer patients after chemotherapy revealed by rs-fMRI (22). Moreover, decreased nodal centralities (reflecting the importance of regions in the brain) were identified in the prefrontal and subcortical regions of brain functional network in post-chemotherapy lung cancer patients (23). All these findings suggested that rs-fMRI might enrich our understanding of the central neural mechanisms underlying CICI and provide a potential and effective method for detecting CICI in lung cancer patients receiving chemotherapeutics (24). At present, regional homogeneity (ReHo), a parameter of rs-fMRI, is often applied to evaluate the neural activity coherence of a given voxel with its nearest neighbors in the brain, which measures the temporal homogeneity of brain activity (25). A previous rs-fMRI study showed that decreased ReHo values were found in the bilateral frontal regions of patients with lung cancer after chemotherapy (26). However, the functional brain alterations underlying CICI following chemotherapy treatment in NSCLC patients, especially divergences between central pathological mechanisms caused by distinct chemotherapeutic drugs, remain somewhat controversial.

This study aimed to explore the functional changes of the brain and associated cognitive impairment in NSCLC patients receiving different chemotherapy regimen. Based on the findings of previous studies, we hypothesized that NSCLC patients after chemotherapy might had impaired brain activities in cognition-related brain regions, which might be also related to CICI of patients. Additionally, different chemotherapy regimens might exhibit differences in the central pathological mechanisms underlying CICI. Therefore, we collected the cognitive scale sores and MRI data from NSCLC patients receiving pemetrexed plus carboplatin chemotherapy (PeCC) and paclitaxel plus carboplatin chemotherapy (PaCC), as well as healthy controls (HCs). Differences of brain activities between patients receiving different chemotherapy regimen were compared by the measure of ReHo and their relationships with CICI were also explored in the patient group.

## Materials and methods

### Participants

**Figure 1:**
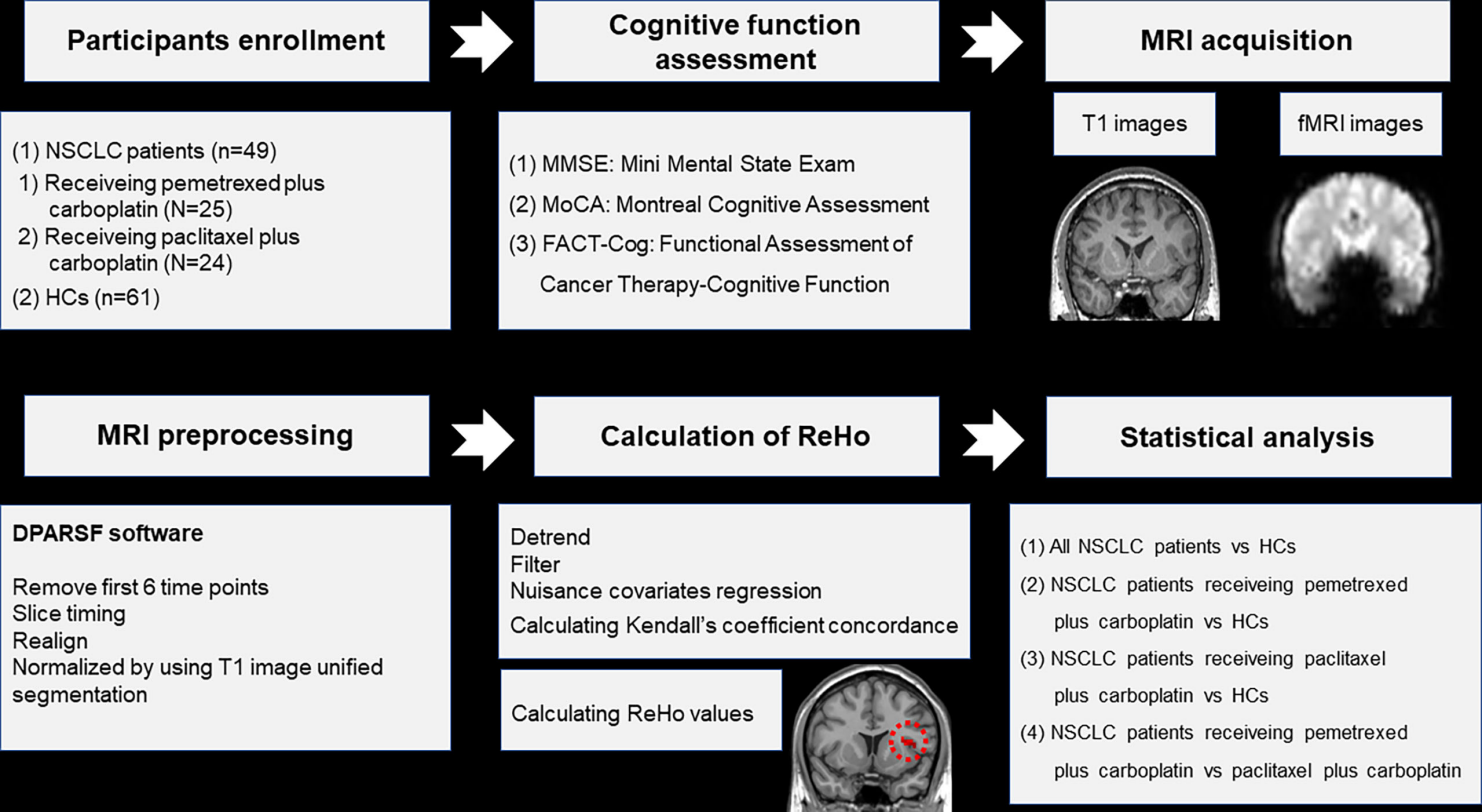
Schematic overview of the study. NSCLC: non-small cell lung cancer; HCs: healthy controls. ReHo: regional homogeneity. fMRI: functional magnetic resonance; DPARSF: data processing assistant for rs-fMRI.

This study was approved by the Ethical Commission of Jiangsu Cancer Hospital & Jiangsu Institute of Cancer Research & The Affiliated Cancer Hospital of Nanjing Medical University. All participants were informed of the experimental protocol (**Figure 1**) and signed informed consent before participation.

In this study, NSCLC patients were recruited from the Department of Oncology, Jiangsu Cancer Hospital & Jiangsu Institute of Cancer Research & The Affiliated Cancer Hospital of Nanjing Medical University, Nanjing, China. All patients were histologically diagnosed of NSCLC (adenocarcinoma or squamous cell carcinoma) without any other cancer. These patients had received 2 to 3 months of standard chemotherapeutic agents (PeCC or PaCC) in accordance with the National Comprehensive Cancer Network (NCCN) guidelines for NSCLC (27) and had no history of radiation treatment. Additionally, age, handness and education level-matched HCs with no history of any cancer were recruited from the local community through advertising. All NSCLC patients and HCs met the following inclusion and exclusion criteria.

The inclusion criteria were as follows: (1) were lower than 70 years old; (2) were right-handed; (3) had received more than 9 years of education. The exclusion criteria were as follows: (1) had abnormal brain structure including brain metastasis; (2) had any severe medical illness; (3) had history of psychiatric or neurological disorders; (4) had history of alcohol or drug abuse; (5) had any contraindication of MRI scans.

To evaluate the cognitive function, all participants underwent a battery of neuropsychological assessments including the Mini Mental State Exam (MMSE) (28), Montreal Cognitive Assessment (MoCA) (29) and Functional Assessment of Cancer Therapy-Cognitive Function (FACT-Cog) (30). The average duration of the cognitive testing was about 30 minutes.

### MRI data acquisition, preprocessing and ReHo calculation

The details about parameters of MRI acquisition and steps of MRI preprocessing were described in our previous study (31), as well as the software applied in the preprocessing (**Figure 1**). The preprocessing steps were as follows: (1) removal of first 6 time points; (2) slice timing; (3) realign; (4) normalized by using T1 image unified segmentation. The measure of ReHo was calculated by the software of DPARSF (32) (**Figure 1**). The steps of ReHo calculation were as follows: (1) detrend; (2) filter; (3) nuisance covariates regression; (4) calculating Kendall’s coefficient concordance and ReHo values of 90 regions in the anatomical automatic labeling (AAL) template (33) (**Figure 1**). Participants were excluded if their head motion exceeded 2mm translation or 2 rotation in this study.

### Statistical analysis

Firstly, two-sample *t*-test and Chi-squared test were applied to compared the differences of age, educational level, scores of cognitive scales and gender distribution between groups by the software of SPSS, respectively. The level of statistical significance was set at *P*<0.05. Secondly, two-sample *t*-test was performed to compare the differences of ReHo values between groups by the software of Resting-State fMRI Data Analysis (REST) Toolkit (34). The corrected level of statistical significance was set at *P*<0.001 with a cluster size>6 voxels using the AlphaSim program. Finally, the relationships between ReHo values of altered brain regions and cognitive scale scores were explored by *Pearson* correlation analysis in the patient group. The significance level was set at *P*<0.05.

## Results

### Comparison of demographic and clinical features

The demographic and clinical characteristics of two groups were presented in **Table 1**. A total of 49 NSCLC patients (adenocarcinoma: 25(51%); squamous cell carcinoma: 24(49%); 0(0%) in stage I, 3(6%) in stage II, 4(8%) in stage III and 42(86%) in stage IV; 25(51%) patients receiving PeCC and 24(49%) patients receiving PaCC) and 61 HCs were enrolled in this study. In all patients, 3(6%) patients had no metastasis while other cases had metastasis in the Lymph gland 13(27%), liver 9(18%), bone 21(43%), as well as intraperitoneal 3(6%).

**Table 1:** Demographic and clinical characteristics of NSCLC patients and HCs.

Variables	NSCLC (n=49)	HCs (n=61)	*t*/χ^2^	*P*
**Age (years)**	60.35 ± 8.58	58.38 ± 6.17	1.40	0.16^a^
**Gender (male/female)**	39/10	42/19	1.61	0.20^b^
**Education level (years)**	13.53 ± 1.82	13.97 ± 1.70	-1.30	0.20^a^
**Cognitive function assessment**				
Scores of MMSE	26.78 ± 1.16	27.28 ± 1.02	-2.42	0.02^a^
Scores of MoCA	26.57 ± 0.98	27.00 ± 0.93	-2.35	0.02^a^
Scores of FACT-Cog	98.92 ± 5.04	101.57 ± 6.86	-2.26	0.03^a^
**Histological diagnosis: n (%)**				
Adenocarcinoma	25 (51%)	–	–	–
Squamous cell carcinoma	24 (49%)	–	–	–
**Disease stage: n (%)**				
I	0 (0%)	–	–	–
II	3 (6%)	–	–	–
III	4 (8%)	–	–	–
IV	42 (86%)	–	–	–
**Metastasis: n (%)**				
No	3 (6%)	–	–	–
Lymph gland	13 (27%)	–	–	–
Liver	9 (18%)	–	–	–
Bone	21 (43%)	–	–	–
Intraperitoneal	3 (6%)	–	–	–
**Chemotherapy regimen: n (%)**				
Pemetrexed plus carboplatin	25 (51%)	–	–	–
Paclitaxel plus carboplatin	24 (49%)	–	–	–

NSCLC, non-small cell lung cancer; HCs, healthy controls; MMSE, Mini Mental State Exam; MoCA, Montreal Cognitive Assessment; FACT-Cog, Functional Assessment of Cancer Therapy-Cognitive Function. P<0.05 was considered to be statistically significant. ^a^: P values were obtained using two sample t-tests. ^b^: P value was obtained using the Pearson chi-square test.

No differences were found in the age, gender and educational level between groups. However, decreased total scores of MMSE, MoCA and FACT-Cog were found in the patient group when compared with those of HCs (**Table 1**).

### Comparison of ReHo values between groups

**Figure 2:**
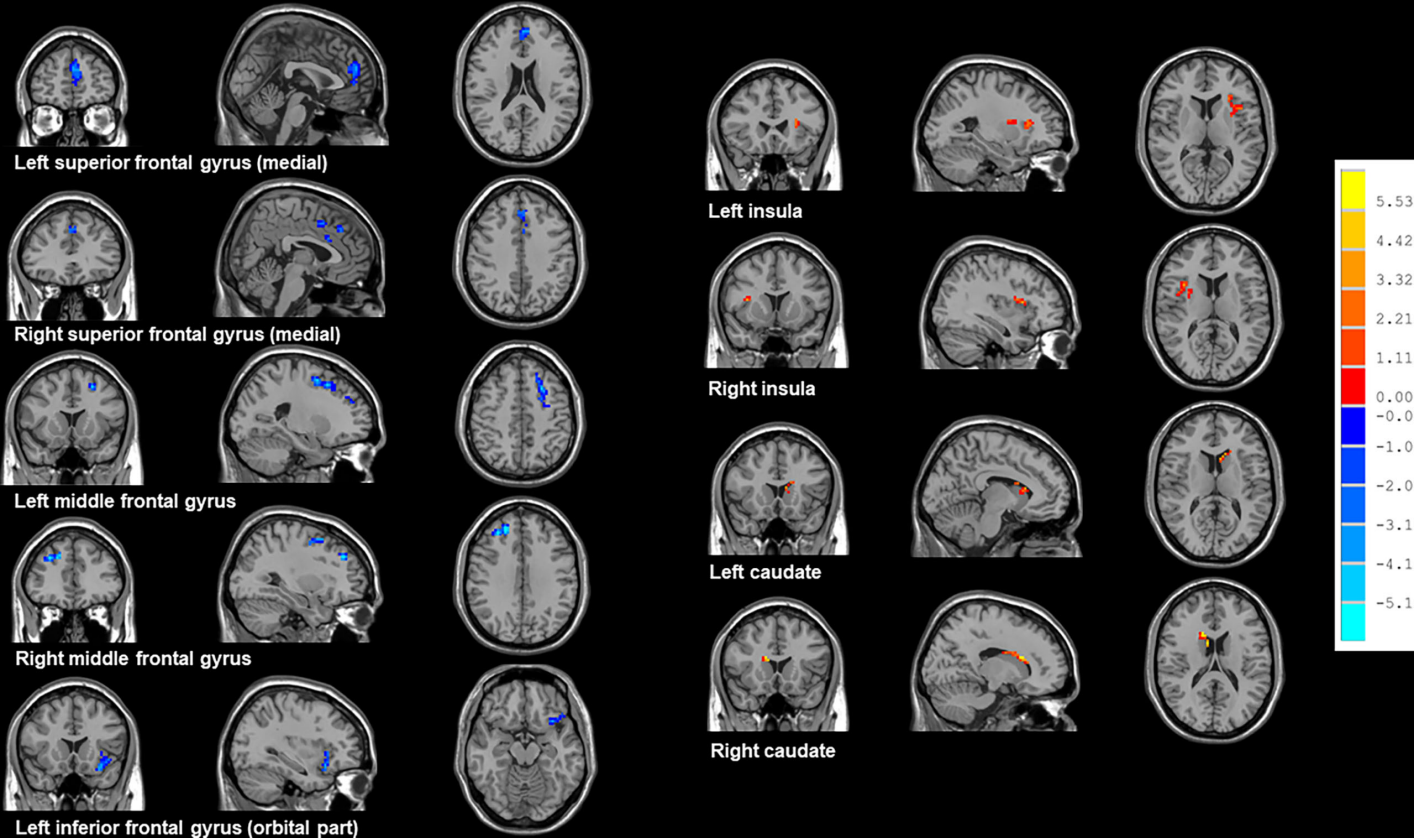
Brain regions showed altered ReHo values between all NSCLC patients and HCs. NSCLC: non-small cell lung cancer; HCs: healthy controls. ReHo: regional homogeneity. The results were obtained by two-sample *t*-tests. The significance threshold was set at *P*<0.001 at the voxel-level and *P*<0.05 at the cluster-level and were corrected for multiple comparisons using Gaussian Random Field (GRF) theory with a minimum cluster size of 36 voxels (two tailed).

As shown in **Table 2** and **Figure 2**, NSCLC patients showed decreased ReHo values in the bilateral superior frontal gyrus (medial), middle frontal gyrus, left inferior frontal gyrus (orbital part) and increased ReHo values in the bilateral insula and caudate.

**Table 2:** Comparisons of ReHo values of brain regions in the AAL template between all NSCLC patients and HCs.

Brain regions	Peak MNI coordinates			Clusters	Peak T values
	x	y	z		
Left superior frontal gyrus (medial)	0	51	21	108	-5.23
Right superior frontal gyrus (medial)	3	33	39	114	-4.75
Left middle frontal gyrus	-24	15	48	147	-5.42
Right middle frontal gyrus	30	39	33	219	-6.19
Left inferior frontal gyrus (orbital part)	-33	18	-15	80	-5.16
Left insula	-27	24	9	63	4.89
Right insula	36	12	12	41	5.10
Left caudate	-9	18	12	38	5.54
Right caudate	15	18	18	50	6.63

NSCLC, non-small cell lung cancer; HCs, healthy controls; ReHo, regional homogeneity; AAL, anatomical automatic labeling; MNI, montreal neurological institute; x, y and z: coordinates of peak voxels of clusters in the MNI space. Peak T values were obtained by two-sample t-tests. The significance threshold was set at P<0.001 at the voxel-level and P<0.05 at the cluster-level and were corrected for multiple comparisons using Gaussian Random Field (GRF) theory with a minimum cluster size of 36 voxels (two tailed).

**Figure 3:**
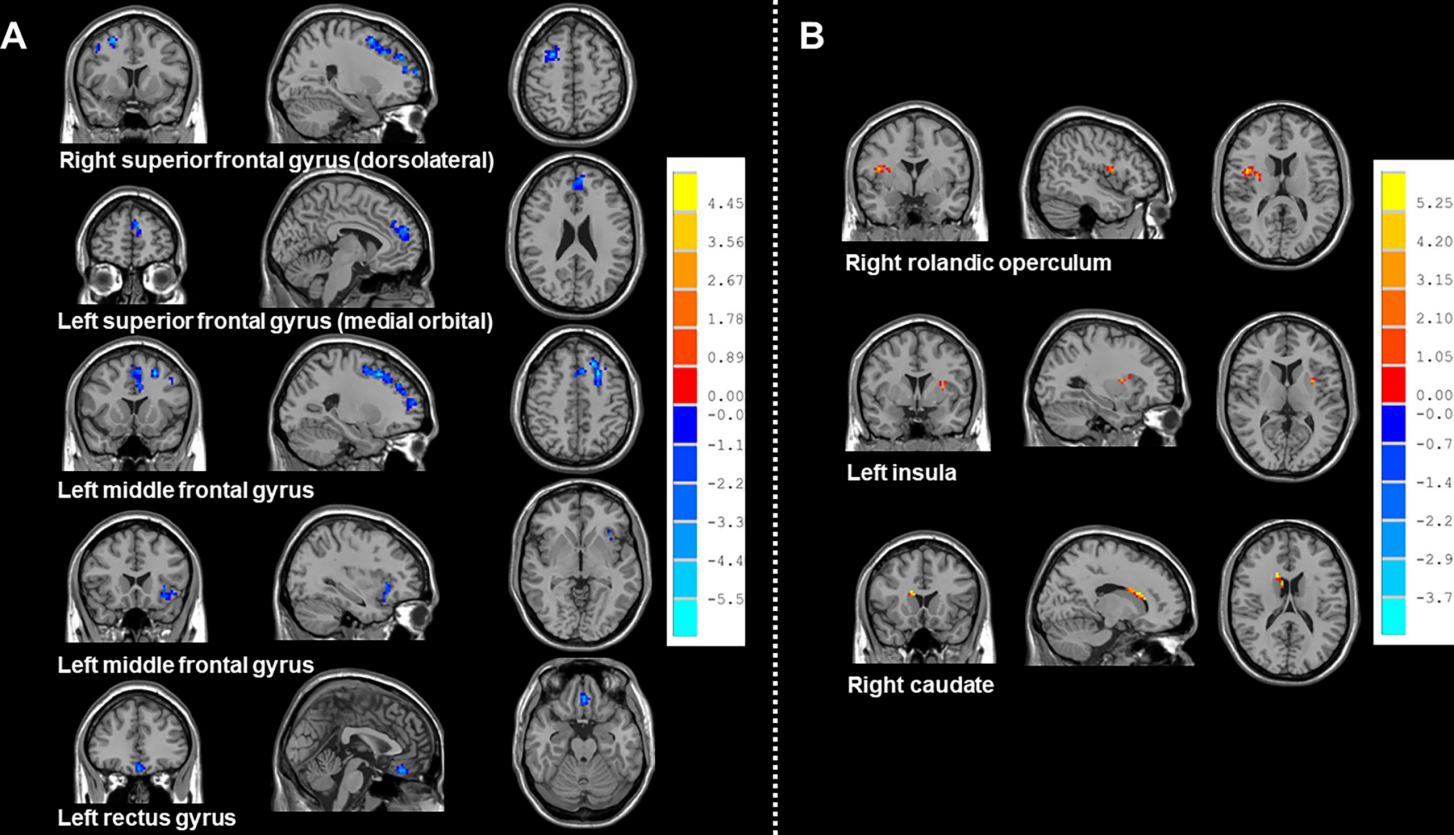
Brain regions showed altered ReHo values between NSCLC patients received pemetrexed plus carboplatin chemotherapy, paclitaxel plus carboplatin chemotherapy and HCs. NSCLC: non-small cell lung cancer; HCs: healthy controls. ReHo: regional homogeneity. **(A)** Brain regions with altered Reho values between NSCLC patients received pemetrexed plus carboplatin chemotherapy and HCs. The results were obtained by two-sample *t*-tests. The significance threshold was set at *P*<0.001 at the voxel-level and *P*<0.05 at the cluster-level and were corrected for multiple comparisons using Gaussian Random Field (GRF) theory with a minimum cluster size of 38 voxels (two tailed). **(B)** Brain regions with altered Reho values between NSCLC patients received paclitaxel plus carboplatin chemotherapy and HCs. The results were obtained by two-sample *t*-tests. The significance threshold was set at *P*<0.001 at the voxel-level and *P*<0.05 at the cluster-level and were corrected for multiple comparisons using Gaussian Random Field (GRF) theory with a minimum cluster size of 26 voxels (two tailed).

Compared with HCs, patients receiving PeCC demonstrated decreased ReHo values in the right superior frontal gyrus (dorsolateral), left superior frontal gyrus (medial orbital), middle frontal gyrus, insula and rectus gyrus (**Table 3**; **Figure 3**) while patients receiving PaCC presented increased ReHo values in the right rolandic operculum, left insula and right caudate (**Table 4**; **Figure 3**).

**Table 3:** Comparisons of ReHo values of brain regions in the AAL template between NSCLC patients received pemetrexed plus carboplatin chemotherapy and HCs.

Brain regions	Peak MNI coordinates			Clusters	Peak T values
	x	y	z		
Right superior frontal gyrus (dorsolateral)	24	9	54	326	-5.66
Left superior frontal gyrus (medial orbital)	-6	54	24	94	-5.39
Left middle frontal gyrus	-24	15	48	486	-6.67
Left insula	-33	21	-3	48	-4.61
Left rectus gyrus	0	36	-21	46	-5.16

NSCLC, non-small cell lung cancer; HCs, healthy controls; ReHo, regional homogeneity; AAL, anatomical automatic labeling; MNI, montreal neurological institute; x, y and z: coordinates of peak voxels of clusters in the MNI space. Peak T values were obtained by two-sample t-tests. The significance threshold was set at P<0.001 at the voxel-level and P<0.05 at the cluster-level and were corrected for multiple comparisons using Gaussian Random Field (GRF) theory with a minimum cluster size of 38 voxels (two tailed).

**Table 4:** Comparisons of ReHo values of brain regions in the AAL template between NSCLC patients received paclitaxel plus carboplatin chemotherapy and HCs.

Brain regions	Peak MNI coordinates			Clusters	Peak T values
	x	y	z		
Right rolandic operculum	46	0	15	46	5.82
Left insula	-30	3	9	32	5.20
Right caudate	15	18	18	43	6.30

NSCLC, non-small cell lung cancer; HCs, healthy controls; ReHo, regional homogeneity; AAL, anatomical automatic labeling; MNI, montreal neurological institute; x, y and z: coordinates of peak voxels of clusters in the MNI space. Peak T values were obtained by two-sample t-tests. The significance threshold was set at P<0.001 at the voxel-level and P<0.05 at the cluster-level and were corrected for multiple comparisons using Gaussian Random Field (GRF) theory with a minimum cluster size of 26 voxels (two tailed).

**Figure 4:**
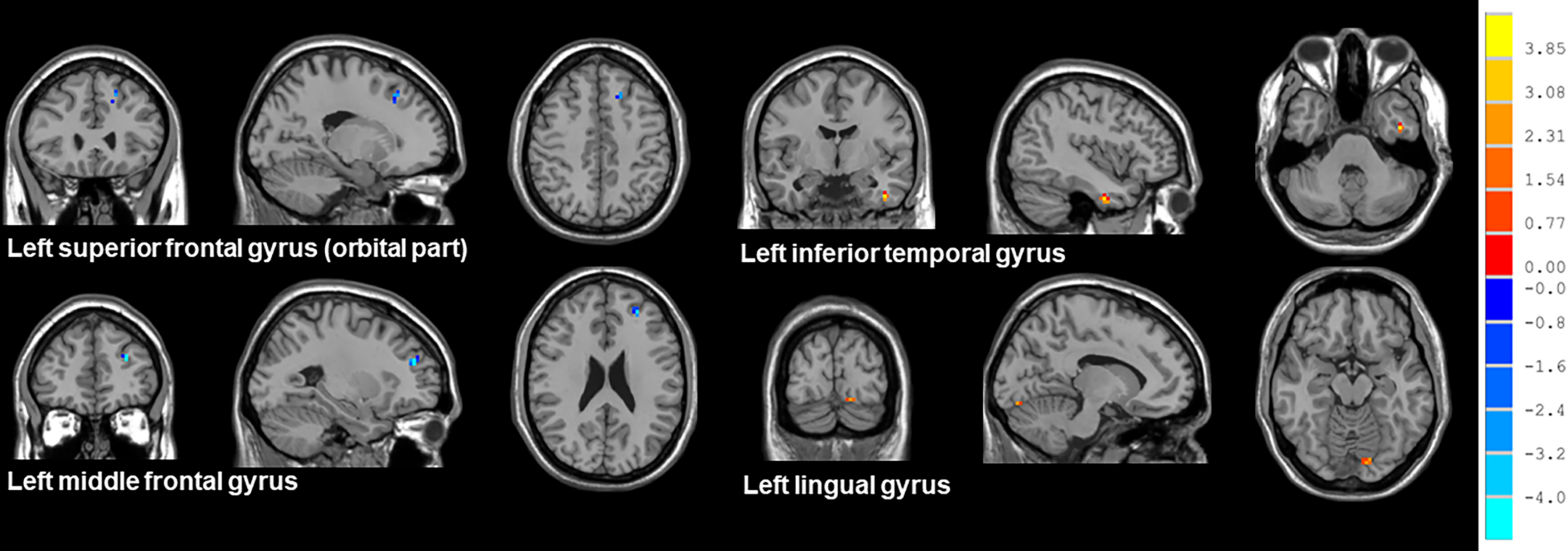
Brain regions showed altered ReHo values between NSCLC patients received pemetrexed plus carboplatin chemotherapy and paclitaxel plus carboplatin chemotherapy. NSCLC: non-small cell lung cancer; HCs: healthy controls. ReHo: regional homogeneity. The results were obtained by two-sample *t*-tests. The significance threshold was set at *P*<0.001 and were corrected for multiple comparisons using the AlphaSim program with a minimum cluster size of 6 voxels (two tailed).

Compared with patients receiving PaCC, patients receiving PeCC had decreased ReHo values in the left superior frontal gyrus (orbital part), middle frontal gyrus and increased ReHo values in the left inferior temporal gyrus, lingual gyrus (**Table 5**; **Figure 4**).

**Table 5:** Comparisons of ReHo values of brain regions in the AAL template between NSCLC patients received pemetrexed plus carboplatin chemotherapy and paclitaxel plus carboplatin chemotherapy.

Brain regions	Peak MNI coordinates			Clusters	Peak T values
	x	y	z		
Left superior frontal gyrus (orbital part)	-18	27	45	7	-4.34
Left middle frontal gyrus	-27	45	24	8	-4.85
Left inferior temporal gyrus	-42	-6	-36	7	4.62
Left lingual gyrus	-12	-87	-15	6	4.11

NSCLC: non-small cell lung cancer; HCs: healthy controls. ReHo: regional homogeneity. AAL: anatomical automatic labeling. MNI: montreal neurological institute; x, y and z: coordinates of peak voxels of clusters in the MNI space. Peak T values were obtained by two-sample t-tests. The significance threshold was set at P<0.001 and were corrected for multiple comparisons using the AlphaSim program with a minimum cluster size of 6 voxels (two tailed).

### Associations between ReHo values of altered brain regions and cognitive scale scores in the patient group

**Figure 5:**
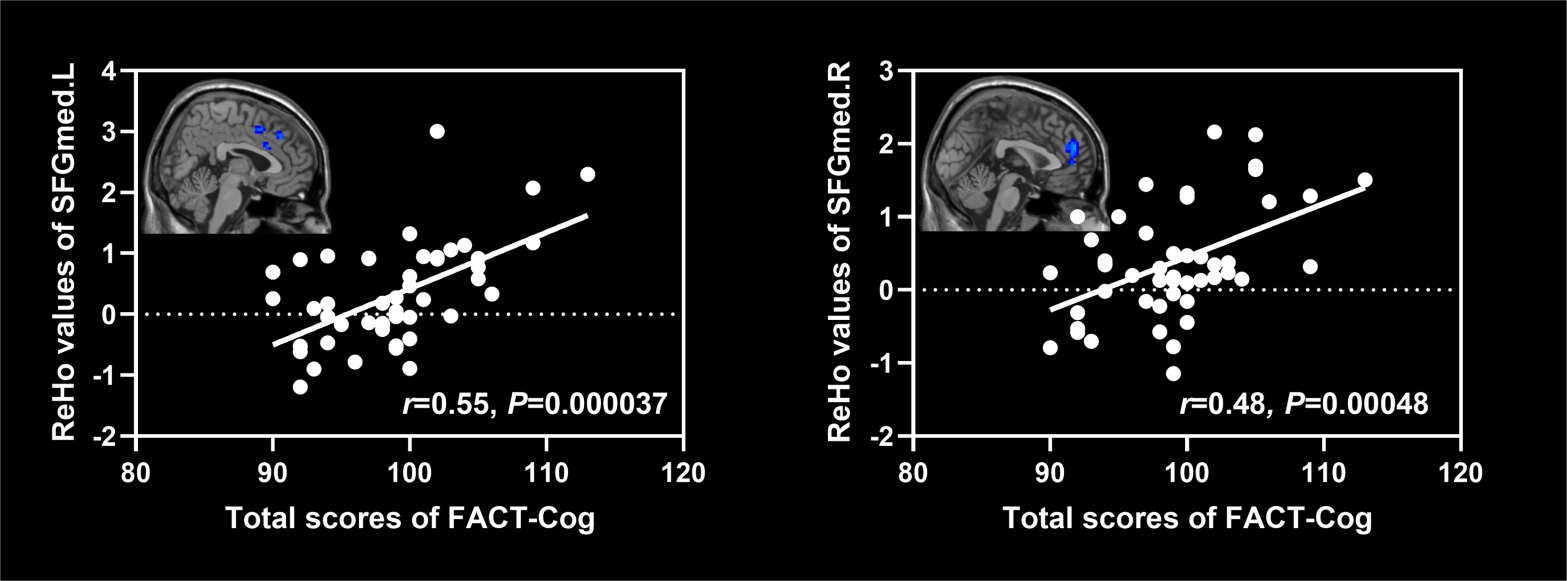
Associations between ReHo values of altered brain regions and scores of cognitive scales in NSCLC patients. NSCLC: non-small cell lung cancer. ReHo: regional homogeneity. SFGmed.L: left superior frontal gyrus (medial); SFGmed.R: right superior frontal gyrus (medial).

As shown in **Figure 5**, ReHo values of the left and right superior frontal gyrus (medial) were positively associated with the total scores of FACT-Cog (*r*=0.55, *P*=0.000037; *r*=0.48, *P*=0.00048).

## Discussion

In this study, we examined functional brain changes and their relationships with CICI in individuals with NSCLC undergone different chemotherapeutical treatment. The findings were as follows: (1) all NSCLC patients undergone chemotherapy showed decreased cognitive function and decreased brain activities in the bilateral superior frontal gyrus (medial), middle frontal gyrus, left inferior frontal gyrus (orbital part) and increased brain activities in the bilateral insula and caudate when compared to HCs; (2) the treatment of PeCC caused decreased brain activities in the right superior frontal gyrus (dorsolateral), left superior frontal gyrus (medial orbital), middle frontal gyrus and insula while PaCC resulted in increased brain activities in the left insula and right caudate; (3) Compared to PaCC, PeCC induced decreased brain activities in the left superior frontal gyrus (orbital part), middle frontal gyrus and increased brain activities in the left inferior temporal gyrus; (4) decreased brain activities of the left and right superior frontal gyrus (medial) were positively related to the CICI of NSCLC patients.

In this study, decreased cognitive function and decreased brain activities in the bilateral superior frontal gyrus (medial), middle frontal gyrus, left inferior frontal gyrus (orbital part) were identified in all NSCLC patients who undergone the treatment of chemotherapy regardless of different types of drugs. The medial prefrontal cortex (mPFC) has abundant connections with other cortical and subcortical regions, which makes it as an essential cortical region for integrating information from other areas and converging updated information to other areas in the brain (35). Take into consideration of its crucial role in the cognitive process including attention, working memory, spatial learning and emotional response, dysfunction of mPFC has been found in various psychiatric and neurological disorders with cognitive impairment (36, 37). Post-chemotherapy lung cancer patients exhibited decreased FC in the left mPFC when compared with non-chemotherapy lung cancer patients (22). The mPFC is one of the most important regions of DMN, which is supposed as a potential biomarker of chemotherapy-related brain injury due to its preferential vulnerability and sensitivity to toxicity and disease states (38). In addition, chemotherapeutic drugs can elevate toxicity in the DMN through several chemotherapy-caused physiologic effects including increased inflammation and oxidative stress (39). Moreover, CICI was found to be associated with cytokine dysregulation and disruptions in neuroplasticity, which presented as disrupted plasticity in mPFC with a striking loss of spines (40). The regional cerebral metabolism of mPFC was found to be correlated with memory complaints of patients following adjuvant chemotherapy (41).

The middle frontal gyrus is the core region of the executive control network, which is involved in cognitive functions, such as executive function, working and prospective memory (42). Abnormal FC pattern was identified in the bilateral superior frontal gyrus and middle frontal gyrus of lung cancer patients (20). Both decreased activation and reduced gray matter volume was found in patients who undergone taxane/platinum (43). Post-CCRT (concomitant chemo-radiotherapy) cancer patients presented decreased FC values between the left middle frontal gyrus and insula (44). In addition, chemotherapy dosage-related cognitive impairment was associated with decreased gray matter density in the right middle frontal gyrus, which played a vital in mediating the chemotherapy dosage effects on verbal fluency performance (45). Moreover, FC values of the right middle frontal gyrus were negatively associated with the executive function of cancer patients after receiving chemotherapy (46). Our finding was consistent with a previous rs-fMRI study, which found decreased average ReHo values in the left middle frontal gyrus and medial prefrontal cortex in patients after completion of first line taxane/platinum chemotherapy (47).

The inferior frontal gyrus (orbital part) is responsible for mediating memories between high-level brain regions including orbitofrontal cortex and insula (48). The inferior frontal gyrus, especially the orbital part, is involved in familiarity judgments, as well as passive perceptual and memory processes (49). Decreased gray matter density was found in the left inferior frontal gyrus and right middle frontal gyrus of patients after chemotherapy treatment with doxorubicin and paclitaxel (50). Decreased ALFF values (a measure reflecting spontaneous neural and cerebrovascular reactivity) were identified in the inferior frontal gyrus, which were associated with poor performance of the cognitive function in cancer patients after chemotherapy (50). In addition, decreased FC values were found between the right inferior frontal gyrus and dorsolateral prefrontal cortex, which were correlated with executive deficits of cancer patients treated with the chemotherapy regimen (docetaxel/adriamycin/cyclophosphamide) (51). Moreover, the cerebral activation and resting metabolism were impaired in the inferior frontal gyrus, which were correlated with performance on memory task in cancer patients with adjuvant chemotherapy remotely (5-10 years previously) when compared with those who had never received chemotherapy (52).

The basal ganglia including caudate is responsible for a variety of cognitive function and plays an important role in planning and execution strategies for achieving complex goals (53). The insula involves in a wide range of cognitive processes, including attention, monitoring the conflict, as well switching between cognitive states (54). Hyperactivity of the left caudate was found in cancer survivors who had finished 3 to 12 months of chemotherapy when compared with those who had not undergone chemotherapy (55). The activation of insula and caudate increased with higher serum methotrexate exposure, which was related to the impairment of cognitive flexibility (56). Smaller gray matter volumes were found in the left caudate of patients treated with chemotherapy during the formative years of brain development, which suggested that systemic chemotherapy had widespread negative effects on brain development (57). However, increased regional grey matter volume was found in the right caudate by the method of voxel-based morphometry in patients had a history of chemotherapy treatment (58). Higher regional cerebral blood flow was identified in the left insula and right caudate in patients with non-small cell lung cancer who had received platinum-based therapy for 3 months to 6 months and altered regional cerebral blood flow connectivity was detected in the bilateral middle frontal gyrus and right caudate (59).

Humans have higher cognitive function and this ability to flexibly organize their behavior, which are regulated predominantly by the prefrontal cortex (60). Damaged function and structure of prefrontal cortex and its associated circuitry are considered to be highly, although not exclusively, associated with cognitive impairment of patients with psychiatric and neurological disorders (61–64). The prefrontal cortex projects to the caudate *via* the frontal-subcortical circuit and the prefrontal-caudate dysconnectivity can contribute to deficits of cognitive performance (65). The impaired prefrontal-caudate circuit may cause damage to the top-down control in the prefrontal cortex and induce compensatory mechanisms in the caudate (66, 67). Individuals with behavioral disorders showed abnormalities of cognitive control networks, especially in parietal regions of fronto-striatal circuit including the prefrontal cortex and caudate, which suggested impaired top-down control of cognitive processes (68). In addition, increased brain activities of one cognitive control network were considered as the compensation in response to reduced brain activities in the other network (68). In this study, PeCC caused decreased brain activities in the right superior frontal gyrus (dorsolateral), left superior frontal gyrus (medial orbital), middle frontal gyrus and insula while PaCC resulted in increased brain activities in the left insula and right caudate. In addition, PeCC induced decreased brain activities in the left superior frontal gyrus (orbital part), middle frontal gyrus and increased brain activities in the left inferior temporal gyrus when compared to PaCC. We speculated that decreased brain activities in the prefrontal cortex might lead to CICI, which might be caused by both PaCC (less severe) and PeCC (more severe) while increased brain activities in the insula and caudate may be a compensation strategy to overcome the imbalanced patterns of brain activity between these regions and associated CICI in patients received chemotherapy, especially PaCC.

## Conclusion

Our results implied that decreased brain activities in the prefrontal cortex might be the central pathological mechanisms underlying CICI while increased brain activities in the insula and caudate might be the compensatory mechanisms for CICI associated prefrontal dysfunction in of NSCLC patients after treatment of chemotherapy. In addition, PeCC might make the abnormal function of the prefrontal cortex more severe while PaCC might lead to more activation of compensatory mechanisms in the insula and caudate. All these provided new insights for better understanding the neurobiological mechanisms underlying CICI in NSCLC patients. However, these findings should be validated by more studies with larger sample size and between patients received different chemotherapy regimen.

## Data availability statement

The raw data supporting the conclusions of this article will be made available by the authors, without undue reservation.

## Ethics statement

The studies involving human participants were reviewed and approved by the Ethical Commission of Jiangsu Cancer Hospital & Jiangsu Institute of Cancer Research & The Affiliated Cancer Hospital of Nanjing Medical University. The patients/participants provided their written informed consent to participate in this study.

## Author contributions

SL, GZ and JF designed the experiments. SL, JN, FY, NY, XL, RM and JW contributed to clinical data collection andassessment. SL, JN and FY analyzed the results. SL, JN and FY wrote the manuscript. All authors contributed to the article and approved the submitted version.

## Funding

The work was supported by the grants of: Jiangsu Provincial Natural Science Fund (No. BK20210977); Cadre Health Research Project of Jiangsu Province (No. BJ18033) and Foundation of Jiangsu Cancer Hospital (No. ZM201923).

## Conflict of interest

The authors declare that the research was conducted in the absence of any commercial or financial relationships that could be construed as a potential conflict of interest.

## Publisher’s note

All claims expressed in this article are solely those of the authors and do not necessarily represent those of their affiliated organizations, or those of the publisher, the editors and the reviewers. Any product that may be evaluated in this article, or claim that may be made by its manufacturer, is not guaranteed or endorsed by the publisher.
